# Beyond End Protection: Shelterin, POT1 Dysfunction, and Telomere Replication Stress in Cancer

**DOI:** 10.3390/biom16091344

**Published:** 2026-09-15

**Authors:** Tadahiko Matsumoto, Akifumi Takaori-Kondo

**Affiliations:** Department of Hematology, Graduate School of Medicine, Kyoto University, Kyoto 606-8303, Japan; tmatsu45@kuhp.kyoto-u.ac.jp

**Keywords:** telomere, POT1, replication stress, telomerase, ALT, cancer

## Abstract

Telomeres (chromosome-end nucleoprotein complexes) are prone to replication fork stalling because of their G-rich repetitive sequences, G-quadruplexes, t-loops, oxidative lesions, and telomeric R-loops generated when telomeric repeat-containing RNA (TERRA) hybridizes with telomeric DNA. In this review, we will summarize how telomeres are replicated and maintained and the mechanisms by which replication stress occurs at telomeres. Subsequently, we will review the role of POT1 mutations in cancer development and pathology and discuss therapeutic strategies targeting telomere biology in cancer.

## 1. Introduction

**Figure 1:**
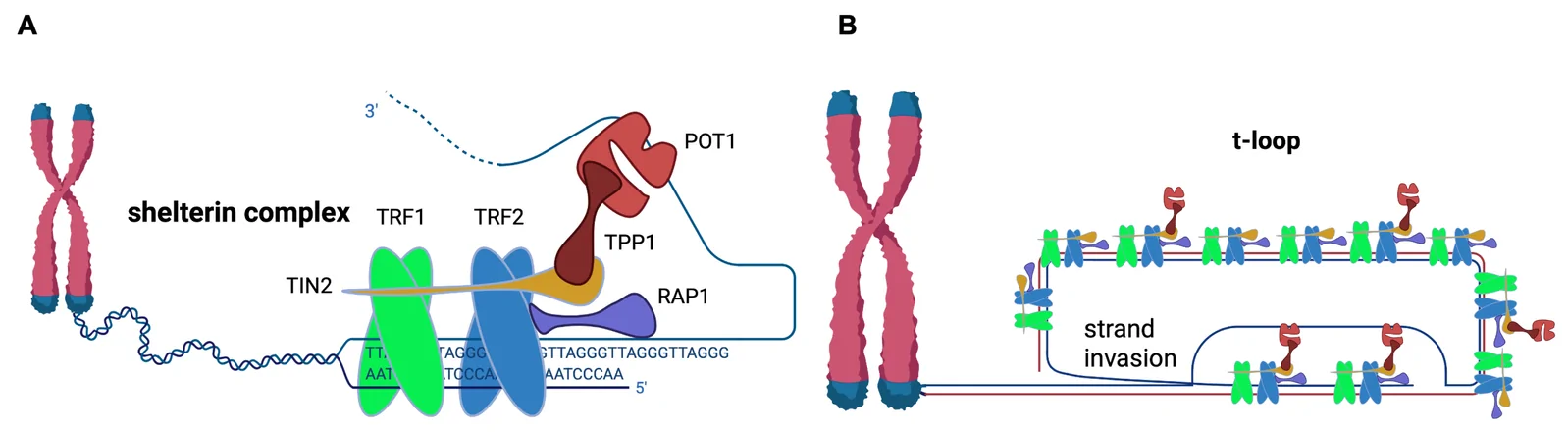
Shelterin complex and telomere architecture. (**A**) Human telomere DNA consists of TTAGGG repeats with a G-rich 3′ single-stranded overhang. TRF1 and TRF2 bind to telomeric dsDNA, and TPP1–POT1 binds indirectly to TRF1 and TRF2 via TIN2. POT1 binds to 3′overhang. (**B**) The 3′ overhang invades double-strand telomeric DNA, generating a loop structure, called as a t-loop, which sequesters the chromosome terminus. TRF2 promotes the formation and stabilization of the t-loop. Created in BioRender. Matusi, H. (2026) https://BioRender.com/m5wxsgl (accessed on 2 September 2026).

**Figure 2:**
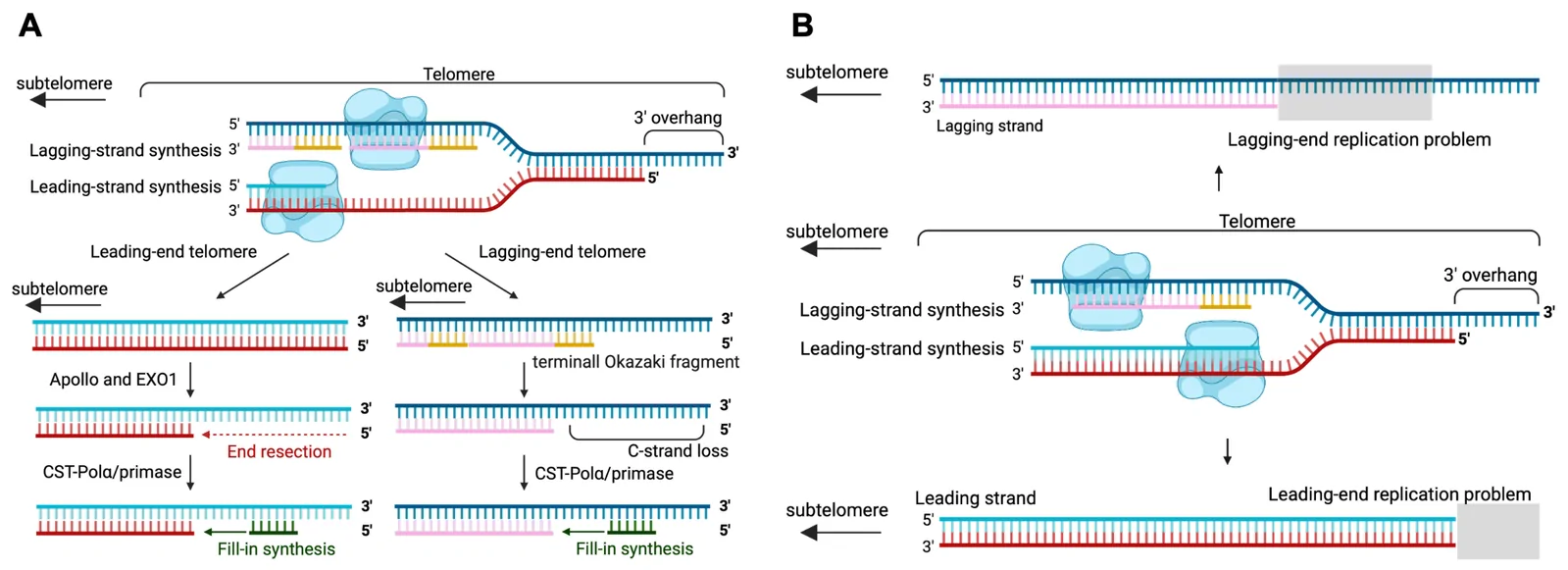
Telomere replication, post-replicative end processing, and the two telomere end-replication problems. (**A**) Replication initiates at a subtelomeric replication origin for conventional telomere replication. Leading-strand synthesis initially generates a nearly blunt end. Apollo, recruited by TRF2, initiates C-strand resection from the 5′ end, with EXO1 contributing further resection, generating a G-strand overhang. At the lagging-end telomere, the terminal Okazaki fragment is primed internally. Removal of its RNA primer leaves a G-strand overhang. The complementary C-strand is incompletely restored by CST–Polα/primase-mediated fill-in. (**B**) Because leading strand is replicated from the shorter C-strand as a template, the resulting leading-strand daughter telomere is shorter. This incomplete replication of telomere is referred to as leading-end replication problem. Incomplete replication of the C-rich strand at the lagging-end telomere constitutes the second telomere end-replication problem and is counteracted by CST–Polα/primase. Template strands are shown in blue and red, nascent leading- and lagging-strand products in light blue and pink, RNA primers in yellow, and CST-dependent fill-in tracts in green. Created in BioRender. Matusi, H. (2026) https://BioRender.com/m5wxsgl (accessed on 2 September 2026).

A telomere is a linear chromosome end structure of DNA and protein complex, which is essential for preventing chromosome ends from being recognized as double-strand breaks [1,2]. Human telomeric DNA consists of a double-stranded DNA (dsDNA) with a 3′ single-stranded overhang (3′ overhang). Moreover, its sequence comprises complete TTAGGG repeats, which range from 5 kb to 15 kb in length. The shelterin complex, which is essential for telomere integrity, is composed of six telomeric proteins (TRF1, TRF2, TIN2, TPP1, POT1, and RAP1) [2,3,4] (Figure 1A). To sequester the chromosome terminus, the 3′ overhang invades into double-stranded telomeric DNA, generating the loop structure, called as t-loop (Figure 1B). Telomeres have been studied mainly in terms of chromosome-end protection and the end-replication problem (Figure 2B). In other words, telomeres prevent natural chromosome ends from being recognized as DNA breaks and suppress inappropriate DNA damage signaling and repair. TRF2 primarily represses ATM signaling and classical non-homologous end joining, whereas POT1 prevents RPA–ATRIP-dependent ATR activation at telomeric single-stranded DNA [5]. Telomeres must also compensate for sequence loss that arises during replication of linear chromosome ends [1,2,6,7,8,9].

Telomeres are also known as genomic regions that are particularly difficult to replicate [10,11,12,13]. In a previous study, TRF1-deficient cells exhibited fragile telomeres (a replication stress marker), indicating that human telomeres exhibit the features of common fragile sites [13]. Based on this finding, telomere abnormalities are thought to be not only the issue of end protection or telomere shortening, but also replication fork stalling during the S-phase (i.e., telomere replication stress). Replication fork stalling or collapse is prone to occur since the telomeric TTAGGG repeat is G-rich and prone to G-quadruplex formation and telomeres form other higher-order structures, including t-loops generated by the invasion of the 3′ overhang and telomeric R-loops generated when TERRA hybridizes with C-rich telomeric DNA [10,11,12,13]. Telomeric oxidative damage and shelterin complex binding to telomeric DNA could be other causes of telomeric replication stress [10,14]. Such telomeric replication stress could result in a fragile telomere, break-induced replication-like reaction, and genomic instability.

From this viewpoint, the shelterin complex is now thought to be not only an end-protection factor, but also a molecular platform that regulates replication stress at the telomere [2,10] (Figure 1A). TRF1 binds to telomeric dsDNA and recruits BLM and, positions TPP1/POT1 heterodimer through TIN2 to relieve telomeric replication stress [15]. Moreover, TRF2 binds to telomeric dsDNA and facilitates t-loop formation to suppress inappropriate activation of the ATM and NHEJ pathways. It also recruits RTEL1 to resolve the t-loop, thereby enabling the replication fork to reach the chromosome end [3]. Among shelterin subunits, POT1 directly binds to the 3′ overhang. POT1 tethered at the telomere excludes replication protein A (RPA), thereby suppressing RPA-ATRIP-dependent ATR activation. Conversely, POT1 loss or dysfunction fails to suppress RPA accumulation at the telomere, leading to ATR signaling activation. In addition, POT1 contributes to C-strand fill-in by recruiting the CST complex and Pol α/primase. POT1 forms a heterodimer with TPP1, positively and negatively regulating telomerase processivity. In particular, recent study revealed that CST and Pol α/primase can compensate for C-strand loss after insufficient Okazaki fragment synthesis at the lagging strand telomere, suggesting that C-strand loss is the second end-replication problem, in addition to end-replication problem arising from G-strand shortening [5,16,17,18] (Figure 2B).

As mentioned previously, the POT1/TPP1 heterodimer plays a critical role in telomere integrity. TPP1 binds to TRF1 via TIN2 (another shelterin component) to recruit POT1 on telomeres and telomerase via protein–protein interaction [2,18,19,20,21]. POT1 binds to the 3′ overhang and suppresses ATR activation. Research has shown that many patients with certain cancers, including chronic lymphocytic leukemia (CLL), melanoma, angiosarcoma, and glioma, harbor POT1 mutations, and the pathological roles of these mutations (e.g., telomere elongation, genome instability, and telomeric replication stress) are attracting attention [22,23,24]. POT1 mutations found in patients with CLL or melanoma have already been reported to be loss-of-function. Most of these cancer-associated mutations induce telomere elongation and varying degrees of telomeric DNA damage. However, which plays a more important role in cancer development remains unclear.

In this review, we will briefly review the mechanisms of telomere replication and elongation and summarize why telomeres are difficult to replicate. Subsequently, replication stress regulation and collapse by the shelterin complex, particularly POT1, and their association with cancer biology will be discussed.

## 2. How Telomeres Are Replicated and Elongated

**Figure 3:**
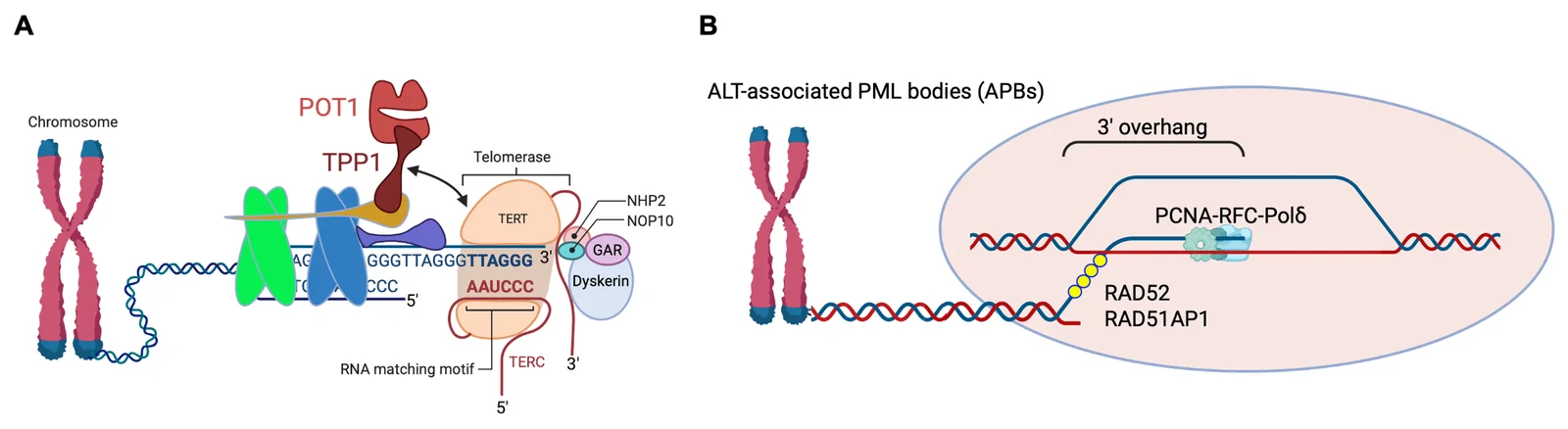
Telomere elongation mechanisms. (**A**) Telomerase is recruited to telomeres through its interaction with the TEL patch of TPP1, as indicated by the double-headed arrow. Telomerase extends the G-rich 3′ overhang by reverse transcription using human telomerase RNA (hTR/TERC) as a template. (**B**) ALT occurs within ALT-associated PML bodies (APBs). The 3′ overhangs of shortened or damaged telomeres cluster within APBs and invade homologous telomeric DNA via RAD51-dependent or RAD51-independent mechanisms. Subsequent BIR-like DNA synthesis mediated by PCNA, RFC, and DNA polymerase δ elongates the telomeres. Created in BioRender. Matusi, H. (2026) https://BioRender.com/m5wxsgl (accessed on 2 September 2026).

Telomere homeostasis relies on semiconservative telomere replication; end processing, including end resection and C-strand fill-in; telomere elongation mechanisms, including telomerase and alternative lengthening of telomeres (ALT) [10,11,12] (Figure 2 and Figure 3). Replication origin firing occurs in the subtelomeric region during normal telomere replication. Moreover, the replication fork proceeds to the chromosome end via telomeric complete TTAGGG repeats during the S-phase. In this process, the leading and lagging strands are synthesized using the C- and G-rich strands as a template, respectively. Given that the C-rich strand is shorter than the G-rich strand, the synthesized leading telomere becomes shorter than that before replication (Figure 2A).

As conventional replication produces structurally distinct daughter ends, 3′ overhang formation and C-strand fill-in synthesis occur sequentially [25,26] (Figure 2A). Initially, leading strand synthesis generates a nearly blunt end. To generate a 3′ overhang, TRF2-bound Apollo initiates the resection of the C-strand from its 5′ end with additional Exo1-mediated resection [26]. By contrast, lagging strand synthesis naturally makes a 3′ overhang. The final Okazaki fragment at the lagging strand is primed internally, and primer processing results in an incompletely replicated region, leading to the formation of the 3′ overhang [25]. After resection or C-strand loss, CST-Pol α/primase performs fill-in synthesis to partially compensate for the loss [19,27,28,29,30] (Figure 2A).

Given that telomere shortening occurs with every replication, a short telomere length is compensated for by telomerase. Telomerase is a ribonucleoprotein complex whose catalytic center comprises TERT and hTR (Figure 3A). hTR is a noncoding RNA, and telomerase retrotranscribes TTAGGG repeats using hTR RNA as a template on the G-strand 3′ end [6]. Telomerase can extend a G-rich 3′ overhang but cannot act on a blunt DNA end. Therefore, at leading-end telomeres, Apollo-initiated resection of the C-rich strand from its 5′ end, with additional resection by EXO1, is required to generate the 3′ overhang that serves as a substrate for telomerase-dependent elongation [26]. Telomerase is recruited to telomeres through its interaction with the TEL patch of TPP1 and extends the G-rich strand [31,32]. Subsequently, the resulting extended 3′ overhang is partially compensated by CST–Polα/primase-mediated C-strand fill-in [19,25,27,28,29,30].

In contrast, some cancer cells utilize a telomerase-independent telomere elongation mechanism called ALT [33,34] (Figure 3B). ALT is not a reaction like telomerase, which synthesizes TTAGGG repeats using an RNA template via retrotranscription. Instead, it is a recombination-dependent DNA synthesis that uses existing telomere DNA on another chromosome [33,34,35,36,37,38]. In human ALT-positive cancer cells, shortened or damaged telomeres accumulate in ALT-associated PML bodies (APBs), and a 3′ overhang invades into telomeric dsDNA in a RAD51-dependent or independent manner. Subsequently, the ALT replisome, which comprises PCNA, RFC, and POLD3, synthesizes long telomeric DNA via break-induced replication (BIR)-like DNA synthesis [33,35,38]. During ALT, several homology-directed recombination intermediates, including strand invasion, D-loop, and Holliday junction, can be formed. However, the BIR-like DNA synthesis plays a central role. BLM, TOP3A, and RMI1/2 form the BTR complex, which contributes to APB formation, ALT replisome assembly, branch migration, G4 resolution, and HR intermediate dissolution, thereby regulating ALT processivity [33,39,40]. Aside from having heterogeneous telomere lengths, APBs, and sister chromatid exchanges, ALT cells contain extrachromosomal DNA with C-rich telomeric repeats (C-circles), which are markers for ALT-positive cells [33,34]. However, whether these extrachromosomal telomeric repeats serve as templates for BIR-like DNA synthesis remains unclear.

As mentioned in this section, telomere replication is a consequence of conventional and semiconservative DNA replication, telomere end processing, telomere elongation by telomerase or ALT.

## 3. Why Telomeres Are Difficult to Replicate

**Figure 4:**
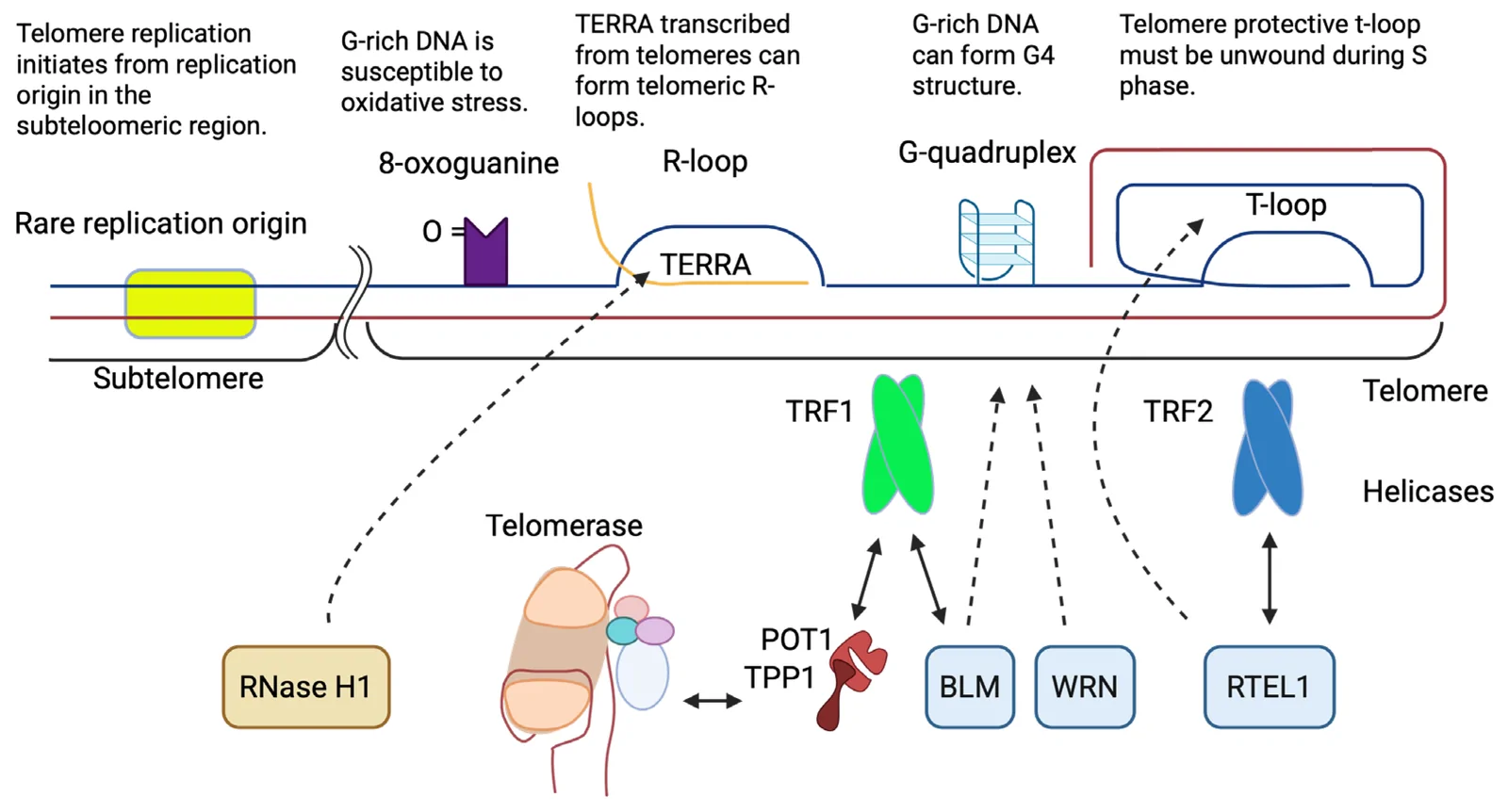
The sources of replication stress at telomeres. Telomeric DNA is G-rich and therefore readily forms G-quadruplex (G4) structures that can impede replication-fork progression. The protective t-loop can likewise serve as a replication barrier and must be transiently disassembled during S phase via TRF2-dependent recruitment of RTEL1. TERRA can hybridize with the C-rich telomeric strand to form R-loops, which are regulated by RNase H1. The G-rich telomeric sequence is also particularly susceptible to oxidative damage, including the formation of 8-oxoguanine (8-oxoG), which can promote replication stress. Because telomere replication generally initiates at subtelomeric origins and replication forks proceed toward chromosome ends, forks stalled within telomeric repeats cannot readily be rescued by converging forks from downstream origins. Shelterin helps coordinate mechanisms that alleviate these replication barriers. TRF1 promotes replication-fork progression by recruiting BLM and, through TIN2, positioning the TPP1–POT1 heterodimer at telomeres, whereas TRF2 recruits RTEL1. RTEL1 promotes t-loop disassembly and helps unwind telomeric G4 structures; BLM and WRN also resolve G4 structures, whereas RNase H1 resolves telomeric RNA–DNA hybrids. Double-head arrows indicate recruitment relationships, and dashed arrows indicate the sources of replication stress counteracted by each factor. Created in BioRender. Matusi, H. (2026) https://BioRender.com/m5wxsgl (accessed on 2 September 2026).

Telomeres are one of the most difficult genomic regions to replicate, and their position (i.e., the chromosome end) is not the reason for the difficulty. The major reasons include their repetitive sequence and highly ordered structures, oxidative damage, and the rarity of replication origins [10,11,12,13] (Figure 4). This idea is strongly supported by Sfeir et al. [13]. The authors found that TRF1-deficient mouse cells show a marked increase in telomere fragility. These fragile telomeres are observed as multiple telomeric signals on metaphase chromosome spreads, resembling the phenotype of a common fragile site under aphidicolin-induced replication stress. This finding indicated that the telomere serves as a barrier to replication-fork progression during the S-phase.

The first reason why telomere replication is difficult is telomeres’ G-rich repetitive sequences, which are prone to G-quadruplex structures [10,41,42]. G4 is a stable secondary nucleotide structure that could obstruct replication fork progression. RTEL1 plays a major role in resolving G4 structures. RTEL1-deficient cells showed an increase in telomere fragility, telomere loss, and T-circle formation, which were exacerbated by a G4 stabilizer. This finding suggests that RTEL1 is crucial for resolving G4 structures and t-loops. Two other RecQ helicases (BLM and WRN) also contribute to G4 resolution during telomere replication [15]. These DNA helicases mitigate replication stress derived from telomeric repetitive sequences.

The second reason is another high-order DNA structure called the t-loop [3]. Telomeres form a loop structure through 3′ overhang invasion into telomeric dsDNA to hide the 3′ end, which is critical to protect the linear chromosome end from inappropriate activation of the ATM or NHEJ pathways. As described previously, RTEL1 also functions as a helicase to resolve the t-loop, and SLX4 cleaves the loop in RTEL1-deficient cells, leading to telomere shortening [43]. Therefore, although the t-loop is required for telomere protection, it must be resolved during telomere replication. Disruption of this regulation results in telomere loss or fragility because of telomeric replication stress.

The third reason is the R-loop, or DNA–RNA hybrid, derived from TERRA (a long noncoding RNA transcribed from repetitive telomere sequences), which reportedly contributes to chromatin regulation and ALT [44,45,46]. In ALT cells, RNase H1 regulates the TERRA–telomeric DNA hybrid and affects telomere length maintenance [44]. Although the R-loop generated by TERRA can provide a scaffold for recombination-dependent telomere elongation, excess amounts of TERRA–telomeric DNA hybrid can induce fork stalling and telomere fragility [38,44,45,47]. TERRA then contributes to telomere integrity and, conversely, causes telomeric replication stress.

The fourth reason is that telomeric DNA is susceptible to oxidative damage because telomeres are G-rich sequences. Guanine is prone to oxidation, which results in the accumulation of oxidative base damage, including 8-oxoguanine. This damage affects telomerase activity and elongation, indicating that oxidative stress involves not only DNA damage but also telomere length regulation and replication stress [10,14]. Taken together, the telomere is a unique region where various replication obstructions, including G4 structures, t-loops, R-loops, and oxidative stress, can coexist.

The fifth reason is the rarity of replication origins around the telomere. In most genomic regions, another replication origin close to the stalled fork rescues stalled replication forks. During telomere replication, the replication origins at each subtelomere fire. Then, the replication forks proceed unidirectionally to each chromosome end [10,11,12]. Thus, a stalled replication fork within the telomere is rarely rescued by replication forks originating from nearby replication origins, leading to a fragile telomere and genomic instability [35,38].

Moreover, recent work has further demonstrated that terminal C-strand synthesis represents a distinct telomere end-replication challenge. At lagging-end telomeres, priming of the Okazaki fragment does not occur on 3′ overhang, leaving the terminal C-rich strand incompletely synthesized after RNA-primer removal. Notably, loss of CST–Pol α/primase results in progressive loss of terminal telomeric DNA from both leading- and lagging-end daughter telomeres after each round of DNA replication. Thus, the second telomere end-replication problem arises from the inability of conventional replication to fully restore the terminal C strand and is counteracted by CST–Polα/primase-mediated fill-in synthesis [30].

In summary, there are multifactorial reasons why telomeres are difficult to replicate, including G4 structures, t-loops, R-loops, oxidative stress, rare replication origins around telomeres, and the second end-replication problem arising from C-strand fill-in. These causes of telomere replication stress can be resolved by the combination of helicases, RNase, and CST-Pol α/primase.

## 4. Shelterin Regulates Replication Stress at Telomeres

Aside from chromosome-end protection, shelterin works as a dynamic machinery that coordinates telomere structure remodeling, telomere replication, end processing, and telomere elongation [2,10,19] (Figure 4). In this section, we will review how each shelterin component regulates telomere replication or elongation. TRF1 supports replication fork progression, whereas TRF2 helps to resolve the t-loop to accomplish telomere replication [13,15,43]. POT1/TPP1 recruits telomerase and CST-Pol α/primase to coordinate G-strand extension and C-strand fill-in [5,19,27,29]. Thus, the shelterin complex acts as a hub that functionally interacts with end protection, replication fork progression, and telomere end processing.

TRF1 primarily promotes fork progression along the dsDNA portion of telomeres [13,15]. Given that TRF1 depletion increases fragile telomeres and that TRF1 helps BLM and TPP1/POT1 localize on telomeres, TRF1 provides a scaffold to resolve replication impediments derived from TTAGGG repeats [15]. Thus, TRF1, which is settled on the telomere dsDNA region, helps the appropriate localization of BLM helicase and the TPP1/POT1 heterodimer, supporting replication fork progression by resolving G4 structures and telomerase recruitment at the 3′ end of the telomere.

TRF2 is required for t-loop formation, which could impede replication fork progression. However, during replication, it, in turn, helps alter the t-loop into a suitable state for replication [2,43]. The t-loop is crucial for restraining the activation of the ATM and NHEJ pathways. On the telomere dsDNA portion, TRF2 recruits RTEL1, which transiently unwinds the t-loop during the S phase to let the replication fork pass through the telomere end [3,43]. TRF2 is also associated with 5′-end resections of the telomere’s leading end, mediated by Apollo, generating a 3′ overhang [19,26]. Thus, TRF2 plays an important role in t-loop formation to protect the telomere from DNA damage response and simultaneously helps smooth telomere replication by facilitating the resolution of end-protective t-loop structure.

POT1/TPP1 is a part of the shelterin complex and binds to telomere ssDNA, which is the 3′ overhang. This heterodimer facilitates telomerase recruitment to telomeres via the TEL patch of TPP1 [48] and increases telomerase processivity in vitro. By contrast, POT1 is thought to inhibit telomerase activity by binding to the 3′ overhang [18,27,29]. This apparent controversy could be explained by the position of TPP1/POT1. POT1 on the internal telomere facilitates telomerase-dependent telomere elongation, whereas POT1 at the 3′ end inhibits telomerase-dependent telomere elongation. Furthermore, a recent study revealed that the CST complex recruited by POT1/TPP1 halts telomerase-dependent telomere extension, which could explain POT1/TPP1’s controversial role in telomere length control. CST, which is recruited by its binding to POT1, stops G-strand extension by halting telomerase and simultaneously promotes C-strand fill-in in coordination with Pol α/primase [27,29]. Therefore, POT1/TPP1 should be regarded as a context-dependent telomere length coordinator that couples G-strand extension by telomerase with C-strand fill-in by CST-Pol α/primase.

Taken together, each shelterin component plays a specific role in telomere replication. TRF1 helps replication fork progression, whereas TRF2 resolves t-loops to make the telomere suitable for extension. TPP1/POT1 regulates telomerase processivity in a context-dependent manner.

## 5. POT1 Mutations in Cancer Patients

**Figure 5:**
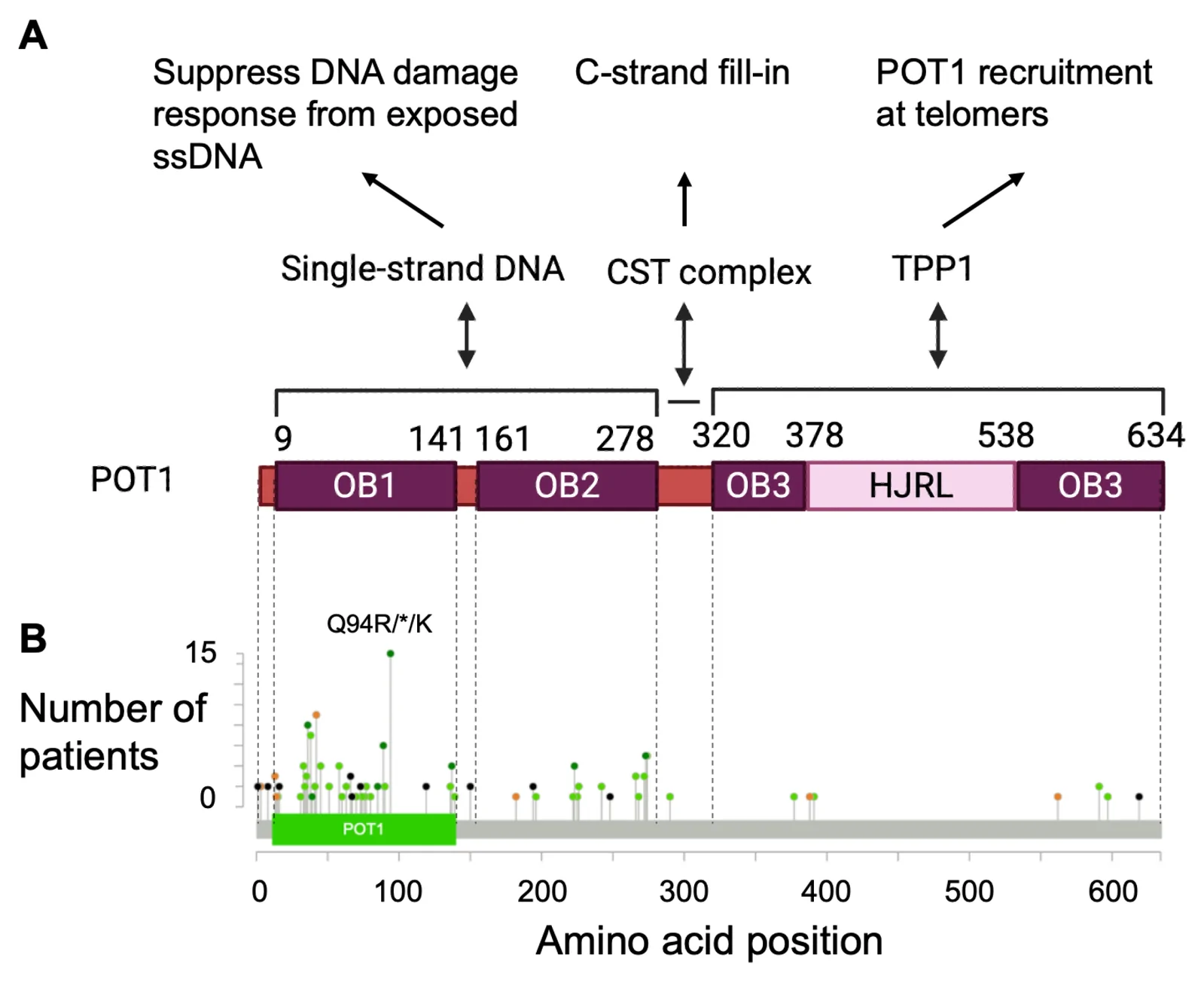
The domains and the molecular functions of POT1 and its mutations associated with cancers. (**A**) Human POT1 is a 634-amino-acid protein. The OB1 and OB2 domains at its N-terminus bind to single-stranded DNA. Its C-terminal split OB3 domain with an inserted HJRL domain binds to TPP1, required for POT1 recruitment on telomeres. The peptide sequence between OB2 and OB3 directly binds to CST complex. Double-headed arrows indicate molecular interactions between individual POT1 domains and the indicated factors. Single-headed arrows indicate the functions of the respective factors. (**B**) The POT1 mutations in mature B cell lymphomas, which are registered in cBioPortal [52,57,58,59,60] (https://www.cbioportal.org, accessed on 29 July 2026). Most of the cancer-associated POT1 mutations are enriched in OB1 and OB2 domains. Green dots indicate missense mutations, black dots indicate truncating mutations, and orange dots indicate splice-site mutations. An asterisk (*) indicates a stop codon. Created in BioRender. Matusi, H. (2026) https://BioRender.com/a26hnu6 (accessed on 2 September 2026).

POT1 is the only shelterin protein that can bind to telomeric ssDNA. Therefore, it plays a central role in 3′ overhang protection, suppression of inappropriate ATR pathway activation, and telomerase regulation [5]. Human POT1 has two OB domains at its N-terminus, which have high affinity to telomeric ssDNA. Meanwhile, POT1 has a split OB-fold domain and a Holliday junction resolvase-like domain at its C-terminus, which is required for TPP1 binding (Figure 5A). Despite POT1’s ssDNA binding capacity, POT1 is recruited not by ssDNA binding but by binding to TPP1, which tethers it to the TRF1/TRF2 shelterin core on telomeric dsDNA via TIN2 [2,49]. Because this tethering increases POT1’s affinity to the telomere, POT1 can competitively suppress RPA binding on the 3′ overhang, resulting in the inhibition of RPA-ATRIP-dependent ATR pathway activation. Unlike humans, mice have two POT1s: POT1a and POT1b. POT1a mainly suppresses ATR pathway activation. Thus, POT1a-deficient cells show telomere elongation, ATR-dependent DNA damage response, and cell proliferation defect. By contrast, POT1b-deficient cells have shorter telomeres and longer 3′ overhangs [2,5,19,26]. These findings indicate that POT1 plays an important role not only in telomere protection but also in telomere end processing.

According to recent reports, POT1 regulates C-strand fill-in, mediated by CST-Pol α/primase [19,25,30] (Figure 5A). It was once thought that human CST is recruited to telomeres through TPP1 binding, whereas mouse CST is recruited by POT1b binding because co-immunoprecipitation (CoIP) experiments failed to detect the CST complex as a human POT1 binding partner. However, a recent cryogenic electron microscopy study revealed that POT1 is the main interactor with CST and uses a conserved interface with mouse POT1b to bind to CST. This study showed that POT1 phosphorylation is required for CST binding, which can explain the contradictory CoIP result [29,50]. This study advocated the CST recruitment model, wherein phosphorylated POT1 binds to inactive CST-Pol α/primase, and its dissociation from POT1 activates CST-Pol α/primase to perform C-strand fill-in. Such C-strand fill-in mediated by CST-Pol α/primase is important to recover C-strand loss, which occurs after lagging-end telomere replication because an Okazaki fragment cannot be synthesized from the very telomere end. This problem is defined as the “second end-replication problem” by Takai et al. Thus, POT1 is required for C-strand integrity through the recruitment of CST-Pol α/primase.

POT1 mutations have been reported in patients with hematological malignancies and solid tumors, and most are thought to be pathogenic [51,52,53]. Initially, these mutations were identified in patient samples, and their pathological roles were validated in vitro, in cells, and in vivo. POT1 mutations are most frequently found in patients with CLL. Next-generation sequencing (NGS) analysis using samples from patients with CLL revealed that POT1 mutations were present in approximately 5% of patients and that most mutations were enriched in the two OB domains at the N-terminus [24,51] (Figure 5B). After investigating the molecular function of those mutations, mutated POT1 proteins were found to have less affinity to telomeric ssDNA in vitro. Moreover, CLL with POT1 mutations exhibited telomere fragility and dysfunction, supporting the view that POT1 mutations are driver, not passenger, mutations. NGS analysis of familial samples of patients with melanoma also detected loss-of-function mutations in POT1, which are associated with melanoma predisposition. Additionally, POT1 mutations were found in patients with Li–Fraumeni-like families without TP53 mutations and patients with familial glioma [22,54]. POT1 R117C mutation (found in Li–Fraumeni-like families) was investigated using an R117C knock-in mouse model [22,54]. This mouse model tends to develop angiosarcoma more frequently than wild-type (WT) mice, and mutant mice have longer telomere lengths than WT mice, although mutant mouse cells exhibit slightly increased levels of DNA damage response. The pathologic significance of these mutations was also analyzed using POT1a conditional knockout (KO) mice [16,17]. Lymphocyte-specific depletion of POT1a and TP53 accelerates T-cell lymphoma development compared with TP53 single KO. POT1a/TP53 double KO cells showed DNA damage at telomeres, including telomere dysfunction-induced foci and ATR activation, increased fragile telomeres, and telomere replication stress. When it comes to mutations that can be related to POT1’s function of CST recruitment, no mutations were reported in the context of cancers. Although POT1 S322L mutation, reported as the cause of Coats plus, impairs CST recruitment, it retains the ability to suppress ATR activation, suggesting that mutation at the POT1–CST interaction surface could affect telomere length control and/or C-strand fill-in [55].

Currently, most cancer-associated POT1 mutations are widely accepted to be loss-of-function [56]. Nevertheless, how they contribute to cancer development remains unclear. POT1 binds not only to telomeric ssDNA as described above, but also to ds-ss DNA junctions to cap the 5′ end of the C-strand telomere with the POT-hole [18]. The telomere C-strand ends with the sequence 3′-CCAATC-5′ because the 5′-terminal phosphorylated Cytosine is held by the POT1 hole, which consists of Y9, R80, H82, and R83 of POT1 [18]. Then, POT1 removal or POT1 loss-of-function mutation disrupts the regulation of telomerase access and CST-Pol α/primase-dependent fill-in, leading to telomere elongation and 3′ overhang lengthening [5]. POT1 R117C mutation results in longer telomeres without increasing DNA damage response, and the longer telomeres enable the cells to proliferate (probably by delaying them from reaching the Hayflick limit) and to survive until they accumulate enough mutations for cancer development [54]. Other POT1a KO mouse models and cellular analyses show that POT1 depletion and cancer-associated POT1 mutations fail to suppress the ATR-dependent DNA damage response, which could induce genomic instability and/or pathogenic recombination [5,16,24,56]. Hence, POT1 mutations could result in telomere elongation and instability, leading to cellular senescence or cell death in checkpoint-intact cells. However, if the cells lack checkpoint function (e.g., TP53 depletion), cells with a POT1 defect proliferate even under replication stress, thereby facilitating the accumulation of genomic instability and promoting cancer development [16].

Cells with a POT1 defect mostly have longer 3′ overhangs. POT1 inhibits telomerase over-elongation because POT1 itself or the CST complex recruited by POT1 binds to the 3′ overhang to conceal the 3′ end as the telomerase substrate [5,18,23,27,31]. However, CST complex recruitment by POT1 is required for appropriate C-strand fill-in [25,29,50]. The POT1 defect cannot suppress G-strand over-elongation and cannot recruit the CST complex for sufficient C-strand fill-in. Thus, this imbalance results in a longer 3′ overhang. This longer 3′ overhang is not only a telomere length abnormality, but also renders the telomere susceptible to RPA-mediated ATR pathway activation and inappropriate recombination. Moreover, it is more likely to have telomeric replication stress derived from longer G-rich sequence. According to a recent study, telomeric replication stress induced by POT1 dysfunction promotes the relocalization of damaged telomeres to the nuclear pore complex [17]. Although it remains unclear what the relocalization of the damaged telomere means and whether this relocalization is required for DNA repair or BIR-like telomere recombination, this relocalization suggests that POT1 mutations may alter not only telomere structure but also nuclear spatial organization, which could contribute to cancer development in cancers with POT1 mutations. Taken together, POT1-mutant tumorigenesis is therefore likely multifactorial, involving ATR-dependent DNA damage signaling, telomere elongation, G-/C-strand imbalance, and altered nuclear organization.

## 6. Telomere Replication and Elongation as a Therapeutic Target

Given that telomere integrity is crucial for the survival and/or proliferation of cancer cells, telomere replication and elongation should be attractive therapeutic targets [6,61,62]. First, a telomerase inhibitor was developed because telomerase-dependent telomere elongation is essential for 80% of overall tumors. Imetelstat is a first-in-class oligonucleotide telomerase inhibitor that binds complementarily to the template region of hTR/TERC and thereby competitively inhibits telomerase activity. It was found to inhibit proliferation or induce apoptosis in cancer and precancerous cells in vitro, suggesting clinical efficacy across many cancer types [63,64]. However, most clinical trials failed to show clinical effectiveness on several types of cancers, including non-small cell lung cancer and neuroblastoma [65]. Nevertheless, imetelstat increased the rate of red blood cell transfusion independence among patients with transfusion-dependent low- to int-1-risk myelodysplastic syndrome (MDS) in the IMerge trial, resulting in approval by the US Food and Drug Administration in 2024 [66,67]. Its mechanism of action is thought to be the elimination of the MDS clone and restoration of normal hematopoiesis.

ALT should be another ideal therapeutic target because ALT is specific to tumors, although approximately 10–15% of cancers have an ALT phenotype [33]. Many intracellular pathways are required for ALT mechanisms, including the ATR pathway, APB formation, RAD51 or RAD52, BIR-like DNA synthesis, and FANCM–BTR pathway. Thus, these pathways could be potent therapeutic targets [33,34,35,38,39,40,68,69,70,71]. As a matter of fact, Flynn et al. reported that ATR inhibition is reportedly effective selectively against ALT-positive cells in vitro [72], whereas Deeg et al. did not observe ALT-specific hypersensitivity to ATR inhibition [73]. RAD52 inhibition was shown to induce cell senescence after long-term culture. RAD52-depleted ALT cells showed decreased DNA synthesis, suggesting that this decrease could be the reason for increased senescence [71]. Moreover, several studies have found that the inhibition of the FANCM–BTR pathway suppresses ALT cells [34,36,37,70]. The toxicity associated with this inhibition should be carefully considered as these pathways could be potent therapeutic targets in ALT-positive cancers.

As described above, among shelterin components, POT1 is the most frequently mutated protein in patients with cancer. Therefore, synthetic lethal therapy for cancers with POT1 mutations could be an attractive therapeutic strategy. So far, no studies have revealed synthetic lethality with POT1 depletion in human cells. Thus, reports using a fission yeast POT1 depletion model should provide useful clues [74]. In fission yeast, Bdf2, Mst1, and Pik1 are required for the survival of POT1-depleted cells [75,76,77]. The depletion of Rqh1 (a RecQ helicase) is synthetic lethal in POT1-depleted cells, and the depletion of RAD51 rescues this synthetic lethality, suggesting that the synthetic lethality is because of inappropriate homologous recombination. Bdf2 and Mst1 are related to acetyl histone metabolism, and they are required for the survival of POT1-deficient fission yeast [77]. Moreover, Pik1 (a Golgi complex-associated PI(4)-kinase) is synthetic lethal with POT1. However, the mechanism behind the synthetic lethality is not related to telomeres [74,76]. The depletion of POT1 in fission yeast generates circular chromosomes. Therefore, these findings in fission yeast could be applied to cells with circular chromosomes more than to POT1-mutated cancers with telomere dysfunction.

## 7. Conclusions

Telomere replication stress plays important roles in the development and pathophysiology of cancer by regulating telomere elongation mechanisms and promoting genomic instability caused by telomere dysfunction (e.g., POT1 loss-of-function mutations). Therefore, these telomere maintenance mechanisms and excessive telomere replication stress, which are preferentially observed in cancer cells, could be attractive therapeutic targets. However, the development of such therapies remains incomplete. As shown by the recent clinical success of imetelstat, targeting telomere elongation and replication stress may provide substantial clinical benefit in certain types of cancer. Therefore, therapies targeting these mechanisms need to be further developed.

## Data Availability

No new data were created or analyzed in this study. Data sharing is not applicable to this article.
